# A randomized trial to evaluate the impact of exercise-based cardiac rehabilitation for the prevention of chemotherapy-induced cardiotoxicity in patients with breast cancer: ONCORE study protocol

**DOI:** 10.1186/s12872-021-01970-2

**Published:** 2021-04-07

**Authors:** Estíbaliz Díaz-Balboa, Violeta González-Salvado, Beatriz Rodríguez-Romero, Amparo Martínez-Monzonís, Milagros Pedreira-Pérez, Patricia Palacios-Ozores, Rafael López-López, Carlos Peña-Gil, José R. González-Juanatey

**Affiliations:** 1 Cardiology Department, Centro de Investigación Biomédica en Red de Enfermedades Cardiovasculares (CIBERCV), University Clinical Hospital of Santiago de Compostela (SERGAS), A Choupana s/n, 15706 Santiago de Compostela, A Coruña Spain; 2 Health Research Institute of Santiago de Compostela (IDIS), A Coruña, Spain; 3 Department of Physiotherapy, Medicine and Biomedical Sciences, Faculty of Physiotherapy, University of A Coruña, A Coruña, Spain; 4 Psychosocial Intervention and Functional Rehabilitation Research Group, Department of Physiotherapy, Medicine and Biomedical Sciences, Faculty of Physiotherapy, University of A Coruña, A Coruña, Spain; 5 Medical Oncology Department and Translational Medical Oncology Group, Centro de Investigación Biomédica en Red de Cáncer (CIBERONC), Santiago de Compostela University School of Medicine, University Clinical Hospital of Santiago (SERGAS), Santiago de Compostela, A Coruña Spain

**Keywords:** Breast cancer, Cardiotoxicity, Cardiac rehabilitation, Exercise, Cardio-oncology, Anthracyclines, HER2 overexpression

## Abstract

**Background:**

Anthracyclines and monoclonal antibodies against human epidermal growth factor receptor-2 (HER2) are frequently used to treat breast cancer but they are associated with risk of developing cardiotoxicity. Implementation of cardioprotective strategies as part of breast cancer treatment are needed. To date, a limited number of studies have examined the effectiveness of cardiac rehabilitation programs or exercise programs in the prevention of cardiotoxicity through an integral assessment of cardiac function. The ONCORE study proposes an exercise-based cardiac rehabilitation program as a non-pharmacological tool for the management of chemotherapy-induced cardiotoxicity.

**Methods:**

The study protocol describes a prospective, randomized controlled trial aimed to determine whether an intervention through an exercise-based CR program can effectively prevent cardiotoxicity induced by anthracyclines and/or anti-HER2 antibodies in women with breast cancer. Three hundred and forty women with breast cancer at early stages scheduled to receive cardiotoxic chemotherapy will be randomly assigned (1:1) to participation in an exercise-based CR program (intervention group) or to usual care and physical activity recommendation (control group). Primary outcomes include changes in left ventricular ejection fraction and global longitudinal strain as markers of cardiac dysfunction assessed by transthoracic echocardiography. Secondary outcomes comprise levels of cardiovascular biomarkers and cardiopulmonary function through peak oxygen uptake determination, physical performance and psychosocial status. Supervised exercise program-related outcomes including safety, adherence/compliance, expectations and physical exercise in- and out-of-hospital are studied as exploratory outcomes. Transthoracic echocardiography, clinical test and questionnaires will be performed at the beginning and two weeks after completion of chemotherapy.

**Discussion:**

The growing incidence of breast cancer and the risk of cardiotoxicity derived from cancer treatments demand adjuvant cardioprotective strategies. The proposed study may determine if an exercise-based CR program is effective in minimizing chemotherapy-induced cardiotoxicity in this population of women with early-stage breast cancer. The proposed research question is concrete, with relevant clinical implications, transferable to clinical practice and achievable with low risk.

*Trial registration* ClinicalTrials.gov Identifier: NCT03964142. Registered on 28 May 2019. Retrospectively registered. https://clinicaltrials.gov/ct2/show/NCT03964142

**Supplementary Information:**

The online version contains supplementary material available at 10.1186/s12872-021-01970-2.

## Background

Survival in breast cancer has improved due to advances in early detection and treatment, leading to reduced breast cancer-related mortality [1]. However, many of the current cancer therapies are linked to multiple side effects. Of these treatments, chemotherapy has been associated with adverse cardiovascular events, such as decreased cardiac function, heart failure, coronary artery disease, arterial hypertension or thromboembolism [2, 3]. In fact, cardiovascular disease is the leading cause of death among women with early-stage breast cancer [4]. The absolute risk of dying from cardiovascular disease in breast cancer ranges from 1.6 to 10.4% [5], and it is higher than that of women from the general population [5, 6]. Treatment using anthracyclines and monoclonal antibodies against human epidermal growth factor receptor 2 (HER2) has resulted in substantial improvements in survival, but these agents have been associated with the development of cardiotoxicity [7, 8]. Cardiotoxicity is defined by either a decrease in left ventricular ejection fraction (LVEF) above 10% from baseline to a value of LVEF under 53%, or a decrease in global longitudinal strain (GLS) deformation below 15% from baseline value [9, 10]. While transthoracic echocardiography (TTE) is the standard method to evaluate cardiotoxicity [9], comprehensive assessment of cardiac function may include other tests, such as biomarkers (troponin I and B-type natriuretic peptide) [11] and cardiopulmonary function measured by peak oxygen uptake (VO2). The latter is considered as a gold standard parameter, which may decrease by 5–26% during exposure to various treatment regimens [12]. The combination of global and specific measures of cardiac function allows identifying patients at risk of heart failure more accurately and initiating appropriate treatment [13, 14]. To respond to this challenge, Cardio-Onco-Hematology (COH) units for early diagnosis and treatment have emerged [12, 15].

Exercise programs are increasingly being recognized as an effective strategy to counteract adverse effects of cancer therapy [16–19]. While there is extensive evidence on the benefits of exercise to mitigate side effects such as fatigue [20] or to improve quality of life [21], evidence on the cardioprotective effects of exercise in breast cancer is still limited [4, 22–24]. In addition to exercise programs, cardiac rehabilitation (CR) programs have proven beneficial to lessen the consequences of cardiovascular disease and reduce the risk of future events [25]. CR programs perform a comprehensive evaluation of cardiovascular risk and a multi-faceted intervention including dietary counselling, weight and blood pressure control, lipid profile optimisation, smoking cessation, psychosocial support, adherence to medical treatment and structured exercise therapy as a central component [26]. Participation in structured CR programs is associated with reduced mortality after an acute coronary event [27], and also with improvement of event-free survival in patients with heart failure [28]. Nonetheless, programs based on CR are not yet standardized within breast cancer care [29].

The main aim of this study is to determine whether an intervention through an exercise-based CR program could effectively prevent cardiotoxicity induced by anthracyclines and/or anti-HER2 antibodies in women with breast cancer at early stages, as compared with usual care and physical activity recommendation. Secondary aims are to determine the effect of the intervention on other cardiac function parameters (cardiopulmonary function assessed by peak VO2 and biomarkers), physical performance outcomes (e.g., body composition, limb strength and upper limb function); and psychometric and lifestyle outcomes (e.g., quality of life, physical exercise, diet pattern). Furthermore, safety of the intervention and supervised exercise program outcomes (adherence-compliance, expectations, satisfaction) are also assessed.

We hypothesize that an exercise-based CR program will be effective in preventing chemotherapy-induced cardiotoxicity in women with breast cancer at early stages, compared to usual care and physical exercise recommendation. Moreover, we hypothesize that patients in the intervention group will maintain or improve their physical performance and psychosocial status.

## Methods

**Fig. 1:**
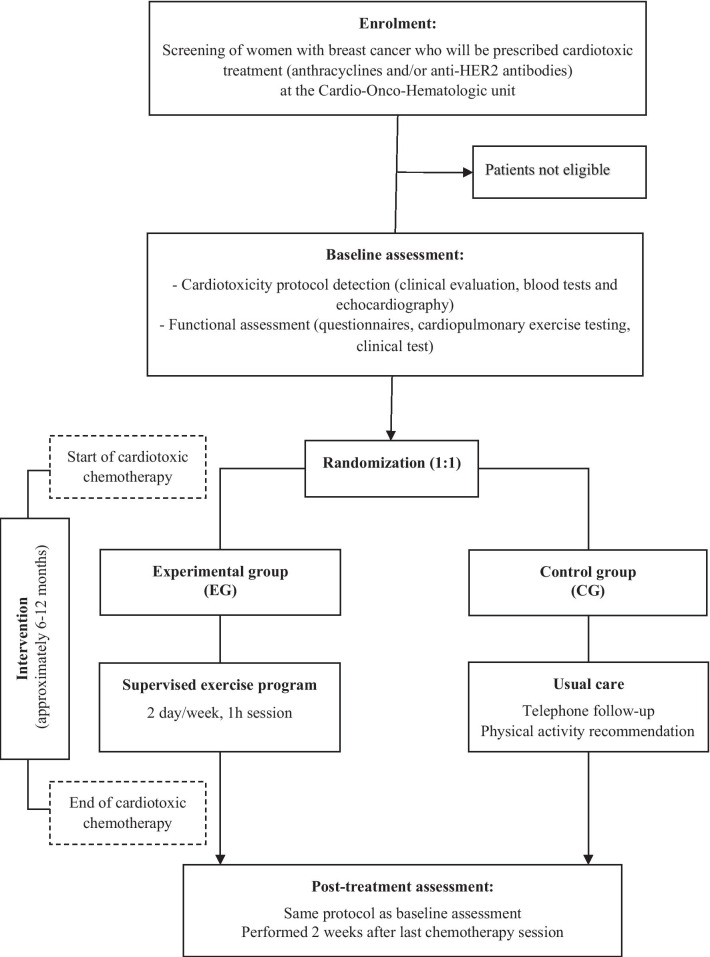
Flowchart ONCORE design

This study protocol follows the SPIRIT 2013 [30] recommendations, an additional checklist file shows this in more detail (Additional file 1). This is a two-arm, prospective, randomized controlled trial comparing an exercise-based CR program (experimental group) to usual care and physical activity recommendation (control group) in women receiving anthracycline and/or anti-HER2 antibodies for early breast cancer. The trial is conducted based on the coordinated work of the COH and the CR and Preventive Cardiology units at the University Clinical Hospital of Santiago de Compostela (Galicia, Spain). The design of the trial is shown in Fig. 1. The study has been approved by the Ethics Committee of Clinical Investigation of Galicia (CEIC reference number 2018/083). Financial support for this work is provided by the Carlos III Health Institute (ISCIII)—PI17/01687, co-funded FEDER, through a competitive grant of the Healthcare Research Fund, under the Strategic Health Action call for proposals.

### Study duration and timing

**Fig. 2:**
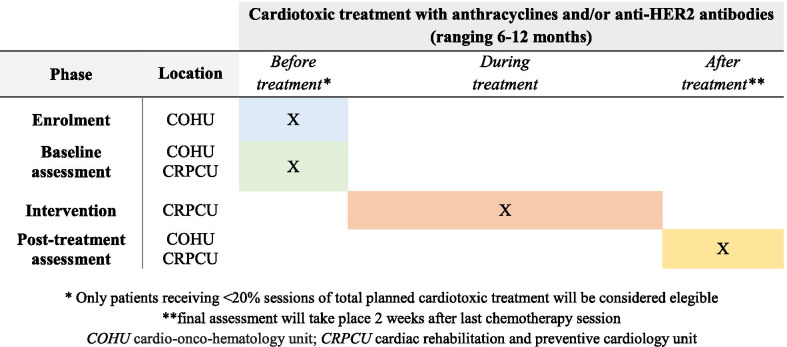
Study timeline

The study timeline is depicted in Fig. 2. Patient recruitment and data collection started in August 2018. The inclusion and baseline assessment of patients is carried out progressively as they start treatment with cardiotoxic chemotherapy including anthracyclines and/or anti-HER2 antibodies, adjusting to the reality of routine clinical practice. The intervention lasts until the last patient recruited finishes the cardiotoxic treatment, between 6 and 12 months, depending on whether this includes anti-HER2 antibodies. Post-treatment assessment is performed two weeks after the last cycle of cardiotoxic chemotherapy including anti-HER2 antibodies. Data collection is expected to be completed in March 2022 when all patients in the study will have completed their respective treatments and undergone post-treatment assessment.

### Recruitment and inclusion

The study population consists of female patients aged between 18 and 70 years with a first diagnosis of breast cancer at early stages (I, II, III), who are planned to receive anthracyclines and/or anti-HER2 antibodies (trastuzumab and pertuzumab), without contraindications to join a CR program or to complete the scheduled visits and providing written consent to participate. If patients are referred once chemotherapy has been initiated, only those who have received less than 20% sessions of the cardiotoxic treatment planned will be eligible for the study. Inclusion and exclusion criteria are detailed in Table 1. Potential participants are detected in daily practice among patients referred to the COH unit to undergo the established protocol to identify cardiotoxicity (clinical, laboratory and echocardiographic follow-up assessment). COH specialists are responsible for initial screening, providing information about the study and offering informed consent to patients.

**Table 1:** Inclusion and exclusion criteria

Inclusion criteria	Exclusion criteria
Woman aged > 18 years and < 70 years	Patients with previous history of heart disease or heart failure
First diagnosis of breast cancer at early stages (I, II, III)	Left ventricular dysfunction (left ventricular ejection fraction < 53%) at baseline
Patients who will receive adjuvant or neoadjuvant treatment with curative intention	Metastatic disease
Treatment with anthracyclines and/or anti-HER-2 antibodies (trastuzumab and pertuzumab)	Patients carrying an implantable cardioverter defibrillator
Possibility of completing a cardiac rehabilitation program and programmed visits	Patients with physical or mental limitation to carry out an exercise program
Providing written informed consent	Refuse to participate
	Overcome 20% of cardiotoxic treatment

### Randomization

After signing the informed consent and undergoing initial assessment, patients are randomized to the experimental group (EG) or the control group (CG) by simple randomization performed by an external person using a computer-generated number sequence. The physiotherapist is responsible for informing each patient of the follow-up modality. The EG will join an exercise-based CR program, while the CG will receive conventional management and physical activity recommendation.

## Intervention

### Experimental group: exercise-based CR program

The intervention in the EG consists of a supervised exercise-based CR program undergone during chemotherapy, which can be administrated before (neoadjuvant) and/or after (adjuvant) surgery. The supervised exercise program takes place at the hospital CR room and is guided and supervised by a physiotherapist, in groups of 7 participants twice a week. Each 1-h session will comprise four parts.

- First part: 15 min of breathing, mobility and balance exercises developed through 8 exercise with 1 set of 7–10 repetitions.
- Second part: 15 min of strength exercises consisting of 4 bodyweight exercises with 2 sets of 7–10 repetitions and 5 resistance bands exercises with 2 sets of 7–10 repetitions.
- Third part: 25 min of continuous or interval exercise in the aerobic-anaerobic transition zone using a cycle ergometer or a treadmill. The patients are monitored with telemetry (Ergoline ERS 2, Germany). A target of 50–85% of heart rate reserve, according to the cardiopulmonary exercise testing (CPET), will be aimed. This objective may be adjusted according to patient’s perceived exertion to rate subjective fatigue values between 3 and 7 (Borg Scale 0–10) and the physiotherapist’s criteria.
- Fourth part: 5 min of flexibilization including 6 types of exercises for the major muscle groups.

Patients in the EG are offered to request an appointment with the CR psychologist if needed. Sessions are held individually for about one hour.

### Control group: usual care and physical activity recommendation

Participants in the CG receive conventional management with general physical activity recommendation. The physiotherapist performs a clinical interview by telephone every two months until final assessment.

### Common intervention for both groups

All patients receive the same supervision within the COH unit, every 2 months since the beginning of the treatment, including cardiotoxicity detection and cardiovascular risk factor control, consisting of dietary advice, smoking cessation, lipid profile, blood pressure control and cardiovascular medical treatment optimization. In addition, a practice guide with exercise recommendations and an exercise routine example is provided, as well as an exercise diary to register physical exercise (including date, minutes and type of exercise done) on a daily basis. Participants in both groups are encouraged and reminded of the importance of adherence to physical exercise.

### Criteria for drop out

Criteria for discontinuing allocated interventions are participant’s will to revoke participation in the study, worsening of oncological prognosis that prevents the continuation and absence of training sessions for unjustified reasons in the case of the EG (below 60% compliance).

### Study outcomes

Outcomes measures and assessment methods are summarized in Table 2. Primary and secondary outcomes are assessed at the beginning and two weeks after completion of chemotherapy. Exploratory outcomes are collected post treatment. The COH specialists perform echocardiographic evaluation and scheduled blood tests. The CR and Preventive Cardiology specialists perform the other evaluation components, including cardiopulmonary function, physical performance and biopsychosocial aspects.

**Table 2:** Study outcome measure

Domain	Measure	Assessment Method	Timepoint from chemotherapy
*Primary outcomes*			
Cardiotoxicity	Resting LVEF	TTE	Pre-post
	Resting GLS	TTE	Pre-post
*Secondary outocomes*			
Cardiopulmonary function	VO2 peak	CPET	Pre-post
Biomarkers	NT- proBNP	Blood test	Pre-post
	Troponin I	Blood test	Pre-post
	Hemoglobin	Blood test	Pre-post
Body composition	% Body fat	Tanita	Pre-post
	% Lean mass	Tanita	Pre-post
Anthropometrics	Height	Height road	Pre
	Weight	Tanita	Pre-post
	Abdominal circumference	Tape measure	Pre-post
	BMI	Database	Pre-post
Vital signs	Heart rate	BPM	Pre-post
	Blood pressure	BPM	Pre-post
Cardiovascular risk factors	Dyslipidemia	Clinical history	Pre-post
	Diabetes mellitus	Clinical history	Pre-post
	Arterial hypertension	Clinical history	Pre-post
	Smoking status	Clinical interview	Pre-post
Limb strength	Lower limb strenght	30-STS	Pre-post
	Uper limb strenght	Handgrip dinamometry	Pre-post
Upper limb function in women undergone surgery	Limb perimeters	Tape measure	Pre and/or post if surgery
	Shoulder functional capacity	Goniometry	Pre and/or post if surgery
	Shoulder pain and disability	SPADI	Pre and/or post if surgery
Psychometric and lifestyle questionnaires	Anxiety and depression	HADS	Pre-post
	Health-related quality of life	FACT-B + 4	Pre-post
	Level of physical activity	GLTEQ	Pre-post
	Adherence mediterranean diet	PREDIMED	Pre-post
*Exploratory outcomes*			
Physical exercise	Minutes of In- (EG) and out- (both groups) of-hospital dedicated exercise	Exercise diary	Post
Safety of the intervention	Adverse events during exercise and clinical serious adverse events	Medical history and reported information	Post
Supervised exercise program	Adherence and compliance	Attendance register	Post
	Expectations	Open question	Post
	Satisfaction	9-item questionnaire	Post

*LVEF* left ventricular ejection fraction, *GLS* global longitudinal strain, *TTE* transthoracic echocardiography, *CPET* cardiopulmonary exercise test, *NT- proBNP* N-terminal brain natriuretic propeptide, *BMI* body mass index, *BPM* blood pressure monitor, *SPADI* shoulder pain and disability index, *PREDIMED* PREvención con DIeta MEDiterránea, *FACT-B* + *4* functional assessment of cancer therapy—breast plus arm morbidity, HADS hospital anxiety and depression scale, *GLTEQ* Godin Leisure Test Exercise Questionnaire, *EG* experimental group

### Primary outcomes

Cardiotoxicity is defined by either a significant decrease of LVEF or GLS, as previously described (9). GLS is included as it is an early and more sensitive indicator of subclinical heart failure, while a decrease in LVEF represents more advanced stages of the disease [10]. The presence of episodes of heart failure will be recorded. LVEF is calculated using Simpson’s biplane rule from the apical 4- and 2-chamber view and GLS is obtained by speckle-tracking analysis of 4-, 3- and 2- chamber view with 2-dimensional echocardiography. Echocardiographic measurements are performed with GE Vivid E95 (GE Vingmed Ultrasound AS, Horten, Norway) ultrasound system by the same experienced operator and doubled checked by a cardiac imaging expert, both of whom are blinded to the clinical data.

A broader assessment of cardiotoxicity includes the full spectrum of cardiovascular complications of cancer treatment and considers other diagnostic options [9, 11], as reflected in the secondary outcomes section.

### Secondary outcomes

#### Other cardiac function outcomes

- Cardiopulmonary function: peak VO2 (ml/kg/min), percentage of predicted peak VO2 (%ppVO2) and metabolic equivalents of task (METs) are assessed by CPET on a continuous incremental test in a cycle ergometer (CardioWise Ergo Fit, Pirmasens, Germany), with workload calculated for each patient depending on body mass, age and fitness.
- Biomarkers: biomarkers of myocardial damage including N-terminal brain natriuretic propeptide (NT-proBNP) and troponin I and hemoglobin (g/dl) are determined on a blood sample.

#### Body composition, anthropometrics and vital signs

Body composition, including % body fat and lean mass, are assessed by electrical bioimpedance (Tanita *MC 780P-MA*). Anthropometrics include assessment of: height, weight, abdominal circumference and body mass index (BMI). Vital signs, including heart rate and blood pressure, are assessed with a blood pressure monitor (Omron M3 IT).

#### Cardiovascular risk factors

The presence of dyslipidemia, diabetes mellitus, arterial hypertension and smoking status are checked on the electronic clinical history and through clinical interview.

#### Limb strength and upper limb function

Lower limb strength is assessed by the number of repetitions in a 30-s sit-to-stand test; and upper limb strength (in kg) is measured in both arms using handgrip dynamometry (Jamar Hydraulic Hand Dynamometer). Upper limb function evaluation in women who have undergone breast surgery or lymphadenectomy is conducted measuring upper limb perimeters for the detection of lymphedema [32]; shoulder functional capacity assessed by range of motion by goniometry [33]; and shoulder pain and disability assessed by the Shoulder Pain And Disability Index (SPADI) questionnaire [34].

#### Psychometric status and healthy lifestyle habits

Anxiety and depression is assessed by the Hospital Anxiety and Depression Scale (HADS) [35]. The Health-related quality of life by the Functional Assessment of Cancer Therapy—Breast plus Arm Morbidity (FACT-B + 4) questionnaire [36]. The level of physical activity is quantified through the Godin Leisure Test Exercise Questionnaire (GLTEQ) expressed as METs/week [37]. Adherence to Mediterranean diet with the PREDIMED (PREvención con DIeta MEDiterránea) questionnaire [38].

### Exploratory outcomes

Physical exercise performed out of hospital by participants in both groups is registered in an exercise diary addressing modality (aerobic, strength, flexibility) and exercise duration (minutes). Additionally, supervised exercise performed in-hospital by participants in the EG is recorded by the physiotherapist.

Safety of the intervention is monitored by the number of adverse events occurred during supervised exercise sessions and other clinical serious adverse events (e.g., anaemia, neutropenia, episodes of heart failure) according to the Common Terminology Criteria for Adverse Events version 4.0 (CTCAE v.4.0) [39].

Adherence to the intervention is defined as the rate of patients completing the program out of those who initiate it, and compliance rate is defined by the number of exercise sessions attended divided by the number of sessions planned. Finally, program-related expectations are assessed at baseline and at the end of the program through an open question ("What do you expect to achieve by participating in the program?”), with responses being categorized into psychological, social and physical spheres. Satisfaction at the end of the program is evaluated by a 9-item questionnaire designed ad hoc.

### Data management

Data is collected electronically through an encrypted online database designed for each study procedure (screening, initial/final assessment, etc.). No identification data is recorded in the database, but an identification number is assigned to each patient. This number is associated to the patient's clinical history number in an independent table only accessible to the ONCORE investigators on a corporate computer. All the information handled is dissociated from the patient's identification information (pseudonymized) and only ONCORE investigators will have access to the identification details.

### Blinding

The type of intervention performed precludes blinding patients or staff involved. Thus, although the professionals involved in the initial assessment (before randomization) are blinded, only those who perform the echocardiogram and the registration of biomarkers are blinded in the final assessment.

### Sample size calculation

Considering the main variable of analysis "development of cardiotoxicity" [9], sample size is determined by comparing two independent proportions to examine the effect of an exercise-based CR program. Based on an expected prevalence of cardiotoxicity in the CG of 20% [31] and 10% in the EG, a variation of 10% between groups is detected, that is, an expected cardiotoxicity prevention of 50% in the EG. The Marrugat et al. [40] formula was used for sample size estimation, accepting an alpha risk of 0.05 and a statistical power of 80%, resulting in the need to recruit 154 participants in each arm. With an expected 10% rate of patients who will not complete the treatment or drop out, it will be necessary to include a total of 340 patients (experimental arm: n = 170; usual care: n = 170).

### Statistical analysis

Analyses will be conducted with SPSS v.25 and R v.4.0.2. software. Both approaches intention to treat and per-protocol analysis will be used. Descriptive analyses will comprise calculating the mean, median, standard deviation, interquartile range for quantitative variables, and absolute and relative frequencies for categorical variables. Distribution and normality of the variables will be determined by one-sample Kolmogorov–Smirnov tests for a sample (significance < 0.05). To detect differences in changes between groups, the data will be analysed using Student's T or ANOVA for parametric variables and with the Wilcoxon test for non-parametric variables. Also effect size will be calculated to see the variance between both groups.

Additionally, correlation and regression analyses will be performed to determine whether the echocardiographic parameters are significantly associated with the changes in cardiopulmonary function, physical performance (e.g., muscle strength), and psychometric status (e.g., quality of life) and lifestyle habits (e.g., diet).

### Dissemination

The trial results will be submitted for publication in a peer-reviewed clinical journal without restrictions and presented at relevant conferences. The investigation will be part of a doctoral thesis project signed by the first author.

## Discussion

This protocol describes a randomized controlled study to examine the effect of an exercise-based CR program on the prevention of chemotherapy-induced cardiotoxicity assessed by echocardiographic parameters (LVEF and GLS), compared with conventional management. Cardiovascular disease is increasing among women with breast cancer and becoming a leading causes of death [4, 12]. Gernaat et al. [5] reported an absolute risk from 1.6 to 10.4% in breast cancer survivors. The increasing use of cardiotoxic treatments, such as anthracyclines and anti-HER2 antibodies, together with cardiovascular risk factors, are the main cause of cardiovascular health worsening. Asymptomatic cardiac dysfunction defined by a subclinical decrease of LVEF, has been associated with a higher incidence of symptomatic heart failure, which occurs in up to 20% of patients depending on treatment regimen [31]. Therefore, other parameters such as GLS, biomarkers or VO2 are considered to increase the sensitivity in the subclinical detection of cardiotoxicity [12].

Coordinated efforts in the early diagnosis of cardiotoxicity within COH units present an opportunity for the study of pharmacological and non-pharmacological strategies to prevent it. Exercise-based CR programs are a ready-to-use resource and can be a valid non-pharmacological treatment strategy, potentially extendable to cancer patients. This network highlights the need for a multidisciplinary group of professionals working in an integrated care process. Exercise-based CR programs offer a comprehensive approach, with cardiovascular risk factors management, psychosocial support and healthy lifestyle counseling. This therapy modality encourages patients to have an active role in their therapeutic process, contributing to increase their perception of control [41].

Exercise, pointed out as a core component of these programs, has been shown to be safe and effective in mitigating side effects of breast cancer treatment, such as worsening quality of life [21], fatigue [20] or decreased physical function [42]. However, several studies have highlighted the need for more evidence on the cardioprotective effects of exercise in preventing cardiotoxicity and improving cardiopulmonary function [4, 12, 29, 43]. Among studies applying an exercise program and evaluating heart function [43–49], broad differences are described for (1) sample size; (2) the period of application of the intervention (during or after treatment); (3) outcomes (echocardiographic parameters, peak VO2, biomarkers, cardiovascular risk factors, etc.); and (iv) evaluation methods (echocardiography, cardiopulmonary exercise testing, indirect estimation of VO2, blood tests, etc.). In addition, some studies [45, 50, 51] and protocols [52–54] have taken into account a broad spectrum of measurements for cardiac function assessment. Kirkham et al. [50] analysed the efficacy of a single aerobic bout performed 24 h prior to each anthracycline treatment in the prevention of breast cancer cardiotoxicity. These authors did not observe a relevant effect of the intervention on the reduction of subclinical cardiotoxicity (echocardiographic or circulating cardiac biomarkers outcomes) but did observe a positive systemic effect comprising hemodynamic adaptations, positive psychological effects, improved body musculoskeletal symptoms and body weight reduction. In turn, a non-randomized trial conducted by Howden et al. [12] examined the cardioprotective effect of a supervised exercise program (2 days/week supervised and 1 day/week unsupervised) in women with breast cancer receiving anthracyclines. They found an attenuation of the decrease in VO2 and higher levels of troponin in the exercise group compared to the usual care group, but no relevant changes were observed in echocardiographic outcomes. Otherwise, in a randomized controlled trial, Hojan et al. [51] compared a 5-day/week exercise program for 9 weeks with usual care in women with HER2 positive breast cancer, noting a protective effect of the exercise program on reducing LVEF and cardiopulmonary function assessed by 6-min walking test, while on plasma parameters they did not find significant differences. Thus, the role of exercise in preventing chemotherapy-induced cardiotoxicity remains unclear. The ONCORE study attempts to elucidate how an exercise-based CR program may contribute to prevent cardiotoxicity in this setting. To this effect, this randomized study includes a larger sample of patients and performs a comprehensive assessment of cardiac function combining TTE, CPET and biomarkers, along with the monitoring of cardiovascular risk factors. In addition, the effects on psychosocial health and physical performance are recorded.

This study has limitations that should be underlined. First, although radiotherapy is also a cardiotoxic treatment [55], it has not been considered as an inclusion criterion alone. This implies that at the end of the study some patients would have received radiotherapy and others would have not. Therefore, radiotherapy sessions received by every patient are recorded for appropriate adjustments. On the other hand, as the overexpression of HER2 occurs in approximately 15–20% of breast cancers [56], the follow-up time in the CR program may differ between patients receiving different treatment schemes, being an expected source of heterogeneity. Given the long duration of the study, especially in patients followed for more than 12 months (HER2 positive), a higher risk of dropout is expected. However, a lengthy intervention duration may benefit the physiological changes induced by exercise adaptations, reflecting a higher effect on echocardiographic and cardiopulmonary function variables. On the other hand, this trial tries to reproduce the reality of clinical practice with all its sources of heterogeneity.

Prevention of cardiotoxicity through an exercise-based CR program would have a high clinical impact [12, 29, 43]. The positive results of this study would be easily transferable to clinical practice in centers that have CR programs, establishing a new treatment indication and providing collaborative support between cardiology, oncology and rehabilitation. The proposed research question is concrete, with relevant clinical implications and achievable with low risk and reasonable investment of resources.

### Adaptations due to COVID-19 pandemic

Due to the situation resulting from the Covid-19 pandemic and to give continuity to the ONCORE project, some amendments are incorporated to guarantee the safety of patients:

- Intervention in the EG: during Covid-19 pandemic, face-to-face supervised therapeutic exercise sessions had to be stopped and telematic supervised exercise sessions are proposed. Exercise in the aerobic-anaerobic transition zone performed on a cycle ergometer or treadmill would be replaced by 14 min interval circuit of 3 series of 5 exercises, with 40 s of work and 10 of rest. This adaptation adds 10 min for strengthening and mobility exercises.
- Experimental group during the pandemic will be a subgroup that would also be analyzed independently.
- Assessment: cardiopulmonary function assessment by CPET would have to be temporarily stopped as disinfection of ventilation systems cannot be ensured. The 6-min walking test (6MWT) would be used to estimate peak VO2 [57].
- Exclusion criteria: a new exclusion criterion would be added: “Patients without possibility to join the telematic exercise sessions”.
- Satisfaction questionnaire: questions related with telematic supervised exercise would be include.

### Trial status

Recruitment for the study commenced in August 2018 and is expected to be completed by March 1st, 2021, considering the withdrawal of one center from the study.

## Supplementary Information

**Additional file 1.** SPIRIT 2013 checklist.

## Data Availability

Data are available upon reasonable request.

## Abbreviations

BMI: Body Mass Index
BPM: Blood pressure monitor
CG: Control group
COH: Cardio-oncohematology
CPET: Cardiopulmonary exercise test
CR: Cardiac rehabilitation
CRPCU: Cardiac rehabilitation and preventive cardiology unit
CTX: Chemotherapy
EG: Experimental group
GLS: Global longitudinal strain
GLTEQ: Godin Leisure Test Exercise Questionnaire
FACT-B + 4: Functional Assessment of Cancer Therapy—breast plus arm morbidity
HADS: Hospital Anxiety and Depression Scale
HER2: Human epidermal growth factor receptor 2
LVEF: Left ventricular ejection fraction
NT- proBNP: N-terminal brain natriuretic propeptide
PREDIMED: PREvención con DIeta MEDiterránea
SPADI: Shoulder pain and disability index
TTE: Transthoracic echocardiogram
